# Metabolic engineering for single-cell protein production from renewable feedstocks and its applications

**DOI:** 10.1007/s44307-024-00042-8

**Published:** 2024-09-29

**Authors:** Zhoukang Zhuang, Guangyu Wan, Xiaocong Lu, Linhai Xie, Tao Yu, Hongting Tang

**Affiliations:** 1 School of Agriculture and Biotechnology, Shenzhen Campus of Sun Yat-sen University, Sun Yat-sen University, Shenzhen, 518107 China; 2 CAS Key Laboratory of Quantitative Engineering Biology, Center for Synthetic Biochemistry, Guangdong Provincial Key Laboratory of Synthetic Genomics, Shenzhen Institute of Synthetic Biology, Shenzhen Institute of Advanced Technology, Chinese Academy of Sciences, Shenzhen, 518055 China; 3 State Key Laboratory of Food Nutrition and Safety, Key Laboratory of Industrial Fermentation Microbiology of the Ministry of Education, Tianjin Key Laboratory of Industrial Microbiology, College of Biotechnology, Tianjin University of Science and Technology, Tianjin, 300457 China

**Keywords:** Food proteins, Single-cell proteins, Renewable material, Metabolic engineering

## Abstract

Proteins are indispensable for maintaining a healthy diet and performing crucial functions in a multitude of physiological processes. The growth of the global population and the emergence of environmental concerns have significantly increased the demand for protein-rich foods such as meat and dairy products, exerting considerable pressure on global food supplies. Single-cell proteins (SCP) have emerged as a promising alternative source, characterized by their high protein content and essential amino acids, lipids, carbohydrates, nucleic acids, inorganic salts, vitamins, and trace elements. SCP offers several advantages over the traditional animal and plant proteins. These include shorter production cycles, the use of diverse raw material sources, high energy efficiency, and minimal environmental impact. This review is primarily concerned with the microbial species employed in SCP production, utilization of non-food renewable materials as a source of feedstock, and application of rational and non-rational metabolic engineering strategies to increase SCP biomass and protein content. Moreover, the current applications, production shortages, and safety concerns associated with SCP are discussed.

## Introduction

Proteins are fundamental building blocks of life and play vital roles in biological reactions, molecular transport, and signal transduction (Rezaei et al. 2016). As projected by the United Nations projections, the global population is expected to reach 10 billion by 2050, necessitating a 50%-80% increase in global food demand (Rischer et al. 2020). To maintain the current level of animal-based protein consumption, the world must produce 1.25 billion tons of meat and dairy products annually (Ritala et al., 2017; Ciani et al. 2021). As living standards improve, the consumer demand for high-quality protein and meat products has increased. However, the expansion of agricultural and livestock production has been constrained by limitations in land availability and environmental concerns. Furthermore, the conversion of plant-to-animal protein is an inefficient process, with a low nitrogen absorption rate from fertilizers and inherent nutrient loss (Berners-Lee et al., 2018). It is therefore evident that relying on traditional agricultural practices alone is unsustainable and inadequate for meeting the growing demand for proteins.

To address future protein shortages, it is necessary to explore alternative sources of protein. A joint report by the Boston Consulting Group and Blue Horizon forecasts that, by 2035, technological advancements and supportive regulations could significantly increase alternative protein consumption from 2% in 2020 to 22%, creating a substantial market valued at approximately $300 billion (Morrison 2022). Approximately one-tenth of the global consumption of meat, eggs, dairy products, and seafood is estimated to be derived from alternative proteins by 2035 (Qin et al. 2022). Four principal categories of alternative proteins are globally recognized: plant, microbial, cell-based, and insect proteins, each derived from distinct raw materials (Rischer et al. 2020; Cunha et al. 2023). These alternative proteins offer more efficient production methods, reducing environmental pollution and resource depletion and mitigating the inherent food safety risks associated with traditional meat products (Choi et al., 2012).

Single-cell proteins (SCP), also known as microbial proteins, refer to the microbial biomass used for protein supply. Protein content is a critical parameter for selecting suitable microorganisms for SCP production. The most commonly used microorganisms include yeast, fungi, algae, and bacteria (Suman. 2015). These organisms typically contain 30%–60% protein by dry cell weight (DCW) (Jach et al. 2022), which is higher than the protein content found in soy, fish, meat, and whole milk (Salazar-López et al., 2022). The concept of SCP gained prominence in the 1960s and the 1970s, a period marked by global food shortages and the pressing need to address protein deficits. During this period, the surplus in global oil production led to the use of oil-based substrates for yeast cultivation, which further drove the development of SCP (Ugalde and Castrillo., 2002). Furthermore, innovative raw materials have been investigated to achieve efficient and cost-effective production of microbial biomass. The term SCP was formally adopted in 1967 during the inaugural international SCP conference at the Massachusetts Institute of Technology (MIT) under the auspices of the United Nations Protein Advisory Group, confirming the potential role of SCP in addressing global food and feed challenges (Frazer 1967). However, concurrent increases in energy prices and global food production have resulted in a temporary decline in interest in the production of edible microbial biomass. As the global population continues to grow and the impact of climate change intensifies, there has been a resurgence of interest in SCP as a novel source of nutrients and proteins (Ciani et al. 2021; Choi et al., 2012). Advancements in synthetic biology have provided a promising avenue for the development of SCPs, as they have enabled the engineering of microbial chassis cells to enhance protein content and diversify protein types using renewable carbon sources. Furthermore, advancements in large-scale microbial fermentation technologies and post-processing methods have established a robust foundation for the future scalability of SCP production (Graham and Ledesma-Amaro. 2023). Consequently, whereas the initial focus of SCP research was on the selection of appropriate strains, current efforts are oriented toward sustainability and the capacity to address global protein supply challenges.

**Fig. 1:**
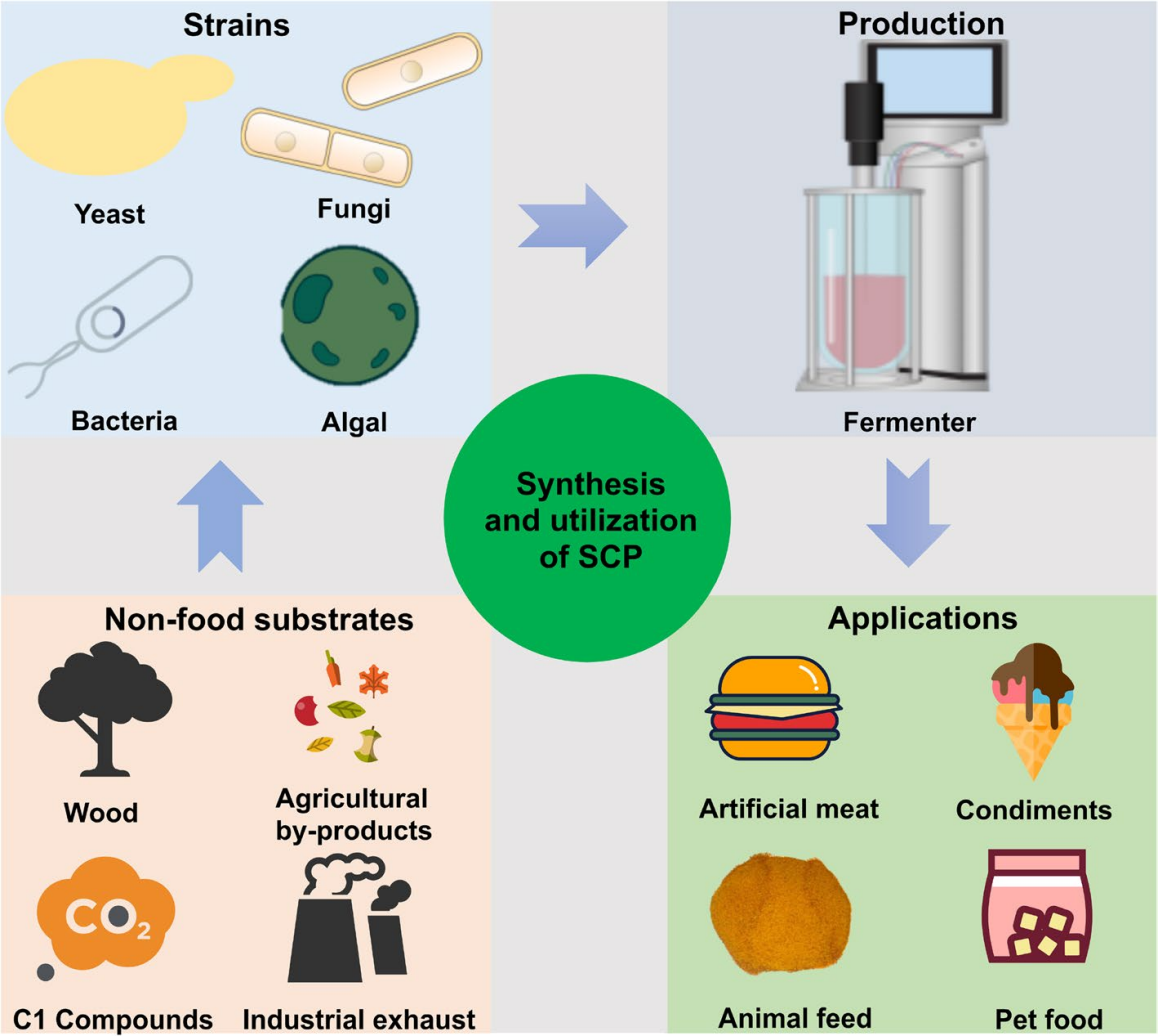
Production of SCP from non-food raw materials and their applications

SCP are distinguished by a cytoplasmic mass that comprises carbohydrates, proteins, fats, nucleic acids, vitamins, and inorganic compounds. Compared with traditional animal and plant proteins, certain SCP achieve yields in bioreactors that can reach several kilograms per liter per hour, which is a significant improvement over traditional agriculture by several orders of magnitude (Ciani et al. 2021). SCP production requires less water and land, is ecofriendly, and does not compete with human food supplies or agricultural land. It is a sustainable protein source that benefits human health and the ecosystem (Bourdichon et al. 2012; Suman 2015; Ciani et al. 2021). SCP has been employed in a multitude of applications, not only as animal feed but also in the development of diverse food products suitable for human consumption (Liu et al. 2022; Qin et al. 2022) (Fig. 1). The replacement of 20% of global beef consumption with fungal proteins by 2050 could cause an annual reduction of over 50% in deforestation (Humpenöder et al. 2022). The use of yeast and its related fermentation products in food production has been a long-standing practice. The most commonly used yeasts for SCP production include *Saccharomyces cerevisiae*, *Kluyveromyces marxianus*, and *Candida utilis*. *Methylotrophic yeasts*, such as *C. utilis*, can produce biomass and proteins from methanol as a carbon source. This process has been successfully scaled up by companies such as Phillips Petroleum, with biomass concentrations reaching 130 g/L and yields exceeding 10 g/L/h (Rashad and W., 1990; Johnson 2013). Given these considerations, it seems reasonable to posit that SCP may play an important role as an alternative protein source.

SCP is an important protein supplement with considerable potential for diverse applications. This review begins by introducing the categories and characteristics of the SCP strains. It then explores non-food renewable materials suitable for SCP production before discussing the applications of rational and non-rational engineering strategies to enhance strain biomass and protein content in SCP. Finally, relevant case studies are examined. Future trends in SCP development are projected and pertinent challenges are addressed.

## Microorganisms used for SCP

Given the accelerated growth of the global alternative protein market, microbial proteins produced through fermentation have garnered attention because of their advantages, including minimal resource consumption, high production efficiency, environmental sustainability, and comprehensive nutrition. The most commonly used microorganisms for microbial protein production include yeast, filamentous fungi, algae, and bacteria (Table 1).

**Table 1:** Applications, protein content, growth substrate, and applications of common SCP microorganisms

Organism	Protein content (%)	Substrate	Applications	References
Yeast	30–60	Industrial and agricultural byproduct, C_1_	Animal feed	(Jach et al. 2022)
Fungi	30–65	byproduct	Alternative meat, egg, Animal feed	(Gamarra-Castillo et al. 2022)
Bacteria	40–80	C_1_ byproduct	food ingredient, Animal, and fish feed	(Hülsen et al. 2022)
Algae	30–75	CO_2_	food ingredient, alternative meat	(Ritala et al., 2017)

*C*_*1*_ One-carbon compounds

### Yeast

Yeast was one of the earliest microorganisms to be used by humans, with historical evidence indicating its use in the production of fermented beverages such as wine and bread (Patrick et al. 2004). Yeast demonstrates robust growth across a broad pH range (2.5–8.5) and temperature range (2–45℃). Yeast typically requires oxygen for optimal growth and its fermentation process can be efficiently scaled up for industrial applications (Jach et al. 2022). Yeast protein is devoid of any essential amino acids, lacks allergenic components, and is free of off-flavors. The protein content of yeast biomass is typically comparable to or higher than that of meat and soybeans, and exceeds that of milk protein. Moreover, the standardized protein efficiency ratio for casein indicates that SCP yeast is comparable to or more efficacious than traditional protein sources (Jach et al. 2022). Most yeast species contain relatively low quantities of nucleic acids, ranging from 5 to 8%. This is advantageous for reducing uric acid accumulation in humans. The long-standing use of yeast in food and beverage applications has contributed to its widespread use. Moreover, yeast can use a range of agricultural industrial by-products, including sugarcane bagasse and potato residues, enhancing the environmental sustainability of yeast production (Jach et al. 2022).

*Saccharomyces cerevisiae* (*S. cerevisiae*) is the most commonly used yeast species in industrial models for biotechnological research. It is generally recognized as safe (GRAS) and has applications in the food, feed, and pharmaceutical industries (Belda et al. 2019; Dunuweera et al., 2021). For example, Marmite®, a Unilever PLC product, has been consumed globally for over a century. Derived from brewing by-products, specifically *S. cerevisiae* extract, Marmite® contains 34% protein of dry cell weight (DCW) and various B vitamins, making it an appropriate dietary supplement for individuals with increased vitamin B requirements (Smetana et al. 2015). *S. cerevisiae* has a protein content between 30 and 55% DCW, high substrate tolerance, and is rich in trace minerals and bioactive compounds, rendering it an attractive feed ingredient that can partially replace products, such as fish meal and soybean meal (Jach et al. 2022).

Another commonly used yeast strain in industrial biotechnology is *Yarrowia lipolytica*(*Y. lipolytica*), which has a protein content ranging from 30 to 50% DCW and a lipid content exceeding 40%. *Y. lipolytica* has been designated as Generally Recognized As Safe (GRAS) by the Food and Drug Administration (FDA), and is regarded as a valuable protein source due to its high essential amino acid content. Since 2010, the dried and heat-killed biomass of *Y. lipolytica* cultivated in biofuel waste has been approved as a feed additive (Berge et al. 2013; Czech et al. 2018). In 2019, the European Food Safety Authority (EFSA) approved the use of dried and inactivated *Y. lipolytica* cells as a novel food ingredient, extending its application as a dietary supplement for individuals aged three and above (JUNCKER, 2019). In 2020, EFSA included selenium-rich *Y. lipolytica* biomass in the list of authorized novel foods owing to its high selenium protein content (Turck et al., 2019). Furthermore, the protein biomass of this yeast represents a valuable source of B-complex vitamins, including vitamin B12 (Jach et al. 2018).

Methylotrophic yeasts include *Pichia pastoris* (*P. pastoris*), *Ogataea polymorpha*, and *Candida boidinii*. These yeasts use methanol as a carbon source via the xylulose monophosphate (XuMP) pathway (Yongjin al., 2020). *P. pastoris* is a prominent industrial methanol yeast that has obtained GRAS certification from the FDA and is extensively used for the production of recombinant proteins (Ahmad et al. 2014; Zhu et al. 2018). Methanolic yeast exhibits a high protein content and is rich in essential amino acids and vitamins. This process effectively converts methanol into high-value SCP, reducing carbon emissions and promoting sustainable development. However, the intricate methanol metabolic pathway and toxicity of intracellular formaldehyde contribute to low methanol utilization efficiency in natural methanol-producing yeasts. Using a combination of elevated fermentation temperatures and adaptive evolution, Meng et al. enhanced the methanol utilization efficiency of *P. pastoris* to 43% (Meng et al. 2023).

### Filamentous fungus

Filamentous fungi are a rich source of nutrients, comprising approximately 45% protein, 25% fiber, 13% fat, and 10% carbohydrates in dry cell weight (DCW), along with a variety of vitamins and minerals (Matassa et al. 2016). Compared with the standards set forth by the Food and Agriculture Organization of the United Nations (FAO), the amino acid profile of filamentous fungi is advantageous. These organisms typically exhibit elevated levels of threonine and lysine while displaying relatively lower methionine content (Ugalde and Castrillo 2002). One of the distinctive advantages of filamentous fungi as microbial protein producers is their capacity to flourish on a diverse array of carbon sources, including fruit by-products, spent grains from beer production, and agricultural residues (Ugalde and Castrillo 2002). This versatility not only enhances their efficiency in protein production but also offers a sustainable solution for the management of waste and by-products from food production and processing. Filamentous fungi are therefore of value not only for their nutritional output but also for their role in the promotion of circular economy principles through the conversion of waste materials into high-quality proteins. The optimal temperature range for fungal growth is 20–30 °C, with a pH range of 5.5–8.0 being conducive to growth. Oxygen is a vital component of the fungal metabolism and growth. The most commonly used filamentous fungi for protein production include *Fusarium venenatum*, *Aspergillus oryzae*, and *Paecilomyces varioti*. For example, fungal proteins derived from *F. venenatum* are employed as suitable substitutes for chicken breast tissue in chicken nuggets, whereas *A. oryzae* contributes to burger patty production (Gamarra-Castillo et al. 2022; Hashempour-Baltork et al. 2023). *F. venenatum* is extensively used for the production of fungal protein. For example, the Marlow Foods SCP product Quorn™ contains approximately 50% protein as determined by DCW (Smetana et al. 2015). This product is primarily used in the production of sausage patties and ready-to-eat burgers. Furthermore, fungal proteins are a source of B vitamins and beneficial secondary metabolites, including β-carotene and ergosterol. The growth of fungi is slower than that of bacteria, particularly for large edible fungi. Most fungi contain nucleic acids at concentrations between 7 and 10% (Li et al., 2023). However, during the cultivation process, toxins produced by some fungi represent a significant concern when using fungi as strains to produce microbial proteins (Anupama & Ravindra, 2000).

### Bacteria

Bacteria have a longstanding history of SCP, with a typical protein content in the DCW range of 50%-80% (Matassa et al. 2016; Khumchai et al., 2022). The essential amino acid content of the bacterial SCP is likely to align closely with the recommendations that the FAO has set forth. Notably, the methionine content in bacterial proteins can reach as high as 3.0%, which is higher than the typical methionine levels found in algal or fungal SCP (Ritala et al., 2017). However, bacterial SCP usually has a relatively high nucleic acid content, ranging from 8 to 12% (Nasseri et al., 2011), which may necessitate additional processing steps to reduce nucleic acid levels to a level suitable for human consumption. Bacterial SCP are produced under both aerobic and anaerobic conditions. Bacteria can thrive in a broad range of temperatures, typically between 15 °C and 45 °C, and require a suitable pH environment, between 6 and 8. Bacterial SCP are distinguished by their rapid growth, with cell mass doubling times ranging from 20 min to 2 h, contingent on the specific strain and cultivation conditions. They can use a diverse range of substrates, including carbohydrates such as starch and sugars, methane, CO_2_, and hydrogen (Pander et al. 2020; Adeoye et al. 2021). The bacteria most commonly employed in SCP production include purple photosynthetic, hydrogen-oxidizing, and methane-used bacteria (FU et al. 2023).

Hydrogen-oxidizing bacteria (HOB), also known as hydrogen–oxygen mixed gas bacteria, use H_2_ and O_2_ as electron donors and acceptors, rapidly fix CO_2_, and assimilate nitrogen for protein synthesis (Matassa et al. 2016). The principal advantage of HOB in microbial protein production is its short production cycle, high protein content (up to 75% of DCW), and amino acid profile, which closely resembles that of high-quality animal protein. As a substantial component of SCP production, HOB uses H_2_ and O_2_ as raw materials generated by renewable energy, offering a potential solution for industries with considerable carbon dioxide emissions to mitigate their environmental impact (Yu, 2018). Furthermore, the HOB SCP contains poly β-hydroxybutyrate, which has been identified as a potential inhibitor of pathogenic bacteria in aquaculture and exhibits probiotic properties (Qin et al. 2022).

Purple phototrophic bacteria (PPB) are facultative anaerobic photosynthetic bacteria capable of metabolic synthesis under both light and dark conditions, using autotrophic and heterotrophic pathways (Capson-Tojo et al. 2020). SCP derived from PPB has a protein content of up to 60% DCW and is enriched with essential amino acids, carotenoids, vitamins, and polyhydroxyalkanoates (PHA), rendering it highly nutritious (Hülsen et al. 2022). The protein powder produced by PPB is predominantly used as a substitute for fish meal in aquaculture feed. During anaerobic fermentation, PPB employs the Calvin-Benson-Bassham (CBB) cycle to fix CO₂; it absorbs near-infrared light through bacteriochlorophylls (BChls) and visible light through its abundant carotenoids (Berg 2011). PPB has been employed in secondary and tertiary wastewater treatments, demonstrating its efficacy in the removal of organic matter, nitrogen, and phosphorus from a range of wastewater sources (Delamare-Deboutteville et al. 2019). However, PPB growth requires adequate illumination, and the cell yield per unit volume frequently remains relatively low.

*Clostridium autoethanogenum* (CA) is an anaerobic chemolithoautotrophic organism that uses CO or CO₂ as a carbon source via the Wood-Ljungdahl pathway and H₂ as a reducing agent to produce fuel ethanol, SCP, and other products (Xing-fa et al. 2020). CA has a protein content exceeding 80% of DCW, with an essential amino acid composition similar to that of fish meal, and is rich in trace elements without anti-nutritional factors (Norman et al. 2018). CA production not only yields a substantial quantity of high-quality protein but also significantly facilitates the recycling of industrial waste gases. Beijing Shougang Technology Co., Ltd., in collaboration with the Feed Research Institute of the Chinese Academy of Agricultural Sciences, has successfully industrialized the production of clean energy, including ethanol and microbial proteins, using CA fermentation technology (Liu et al. 2020). The primary raw material was CO from steel industry gas, the nitrogen source was ammonia water, and other trace elements were added to the medium. This results in an annual industrial production capacity of tens of thousands of tons of biosynthesized SCP (Wan et al. 2024). This approach effectively mitigated carbon emissions and reduced environmental pollution.

Methanotrophic bacteria use methane as both an energy and carbon source, and have been employed for SCP production for decades (Linder 2019). They demonstrated a high growth rate, with a protein content exceeding 70% DCW. Methanotrophic bacteria play a pivotal role in reducing atmospheric methane (CH_4_) levels and mitigating the greenhouse effect (Gao et al. 2024). Several SCP products derived from methane are commercially available. The Danish company UniBio and the American company Calysta have developed fermentation technologies that employ aerobic methanotrophic bacteria via the RuMP pathway to convert methane into SCP on a large scale. *Methylococcus capsulatus* is a promising source of protein (Guo et al. 2022). UniBio has developed UniProtein® through continuous fermentation, resulting in a protein content of 72% of the DCW. The product is distinguished by free-flowing reddish-brown granules with a methane conversion rate of 0.7 g DCW/g, and is devoid of toxins and heavy metals (Øverland et al. 2010). The protein produced by Calysta's *M. capsulatus* is marketed as FeedKind® and displays an extremely high nutritional value, with a protein content exceeding 80% DCW and an annual production capacity of 20,000 tons. *M. capsulatus* is renowned for its nutritional quality, balanced amino acid profile, and high digestibility and absorption rates, rendering it an exemplary source of protein. Research indicates that *M. capsulatus* protein can directly substitute for a portion of high-quality fishmeal in aquaculture feed and can sometimes replace white fishmeal (Zhang et al. 2022).

Besides microorganisms that have been the subject of much discussion, several bacteria that have been designated as Generally Recognized As Safe (GRAS) have been identified as having significant potential as SCP producers because of their safe profiles and efficient protein production capabilities. *Bacillus subtilis* is an aerobic gram-positive soil bacterium that is widely used for heterologous protein production (Earl et al., 2008; Dijl and Hecker, 2013). It is renowned for its high protein yield, which typically ranges from 40 to 60% of DCW. This strain is preferred for SCP production because of its rapid growth and brief fermentation period, typically approximately 48 h (Dijl and Hecker, 2013; Su et al., 2020). *B. subtilis* is a versatile organism capable of utilizing a variety of substrates, including agricultural and industrial byproducts, making it an efficient and sustainable candidate for SCP production. *Corynebacterium glutamicum* is another significant GRAS strain, primarily recognized for its use in amino acid production, but is also a valuable SCP producer. The protein content of this strain is approximately 50%–70% in DCW (Liu et al., 2016). Its metabolic flexibility and genetic tractability render it a promising candidate for metabolic engineering, enabling the production of SCP with tailored amino acid profiles suitable for specific nutritional requirements (Gopinath et al. 2011). Lactobacillus species, which are commonly used in the food industry, can produce SCP with a protein content ranging from 20 to 40% DCW. Moreover, these bacteria can produce beneficial metabolites, such as lactic acid and vitamins, enhancing their utility in the food and feed industries (Dempsey and Corr, 2022).

### Microalgae

Microalgae are photosynthetic microorganisms that use CO₂ as a carbon source and convert it into glyceraldehyde-3-phosphate through the Calvin-Benson-Bassham (CBB) cycle to derive energy from sunlight (Ritala et al., 2017). They serve a variety of purposes as SCP, benefiting from advantages such as straightforward cultivation, utilization of solar energy, rapid growth rates, and high protein content (Yao et al. 2019). Microalgae typically contain approximately 60%-70% crude protein of DCW and have long been considered an effective means of addressing the "protein gap," making them an important source of microbial protein. Microalgae are well known for their highly digestible protein content and balanced amino acid profiles. Additionally, they are rich sources of vitamins, minerals, and polyunsaturated fatty acids (Niccolai et al. 2019). Besides their nutritional value, microalgae have been demonstrated to exhibit a range of bioactivities, including anti-inflammatory, antibacterial, antioxidant, lipid-lowering, and anticancer properties. This makes them a highly attractive subject of study in both nutrition and pharmaceutical fields (Bishop & Zubeck, 2012). Furthermore, microalgae exhibit relatively low nucleic acid levels, typically between 3 and 8% (Nasseri et al., 2011), suggesting their potential for the production of functional high-protein foods (Pereira et al. 2022). Microalgae demonstrate robust growth in a salinity range of 4%-36%, with optimal growth occurring at temperatures of 10–36 °C. They flourished in a neutral environment with a pH of 7.5–8.5. Furthermore, microalgae require light for photosynthesis, with an optimal light intensity of 8,000–15,000 lx. The most commonly used microalgal protein sources, including *Chlorella*, *Chlamydomonas*, *Nannochloropsis*, and *Spirulina*, are predominantly used in supplement formulations. The global market for autotrophic microalgae is approximately 30,000 tons, with an increasing number of applications in foods based on *Spirulina platensis* and *Chlorella* proteins. Recently, *Tetraselmis chuii* and *Chlamydomonas reinhardtii* have been approved as new food resources (Wan et al. 2024). Despite considerable interest among researchers in the biotechnological applications of autotrophic microalgae, commercial-scale production of these organisms remains limited. This is primarily due to the requirement for sufficient light and the substantial energy consumption associated with the process. Therefore, it is imperative to optimize production costs and improve production efficiency if microalgal protein production becomes a commercially viable proposition.

## Non-food raw materials for SCP production

SCP production has traditionally involved the use of conventional substrates, including starch, molasses, and fruits. However, the cited sources do not provide sufficient evidence to support this claim. (Ugalde and Castrillo 2002; Tang et al. 2023). However, these substrates compete with human food and land resources, and are expensive. Therefore, it is necessary to develop non-food substrates, including agricultural and industrial byproducts, kitchen waste, and one-carbon substrates, for SCP production.

### Byproducts of the brewing industry

The solid waste produced by the brewing industry, known as distillery yeast sludge, contains significant quantities of proteins and essential amino acids, rendering it an optimal substrate for SCP production. Valentino et al. investigated the potential of various microorganisms isolated from brewer distillery yeast sludge to produce SCP via the fermentation of dried sludge (Valentino 2015). The highest crude protein content (33.7% DCW) was observed in the *S. cerevisiae* strains inoculated with brewer's sludge, which was significantly higher than that in the non-inoculated strains (25.1%). Furthermore, essential amino acids such as methionine and tryptophan increased considerably, suggesting the potential of brewer distillery yeast sludge as a carbon source for SCP production. In another study, Chai et al. sterilized and disinfected the spent grain of a brewer by fermenting it with *Rhizopus oligosporus*. Following a three-day microbial fermentation, researchers employed microwave-assisted three-phase partitioning bio-separation to extract and separate microbial proteins, obtaining 200 g of protein from one kilogram of feedstock (Chai and Chen 2024). Moreover, the brewing industry generates considerable quantities of solid byproducts and wastewater that can be aerobically treated to facilitate SCP production. In a related study, Lee et al. (2014) investigated the enrichment of diazotrophic microbial communities in brewery wastewater and optimized the conditions for producing SCP, achieving a protein content exceeding 55% DCW. This approach addresses both the solid waste and wastewater issues prevalent in the brewing industry.

### Byproducts of agro-processing industries

The processing of potato starch generates a considerable amount of waste material, which presents a significant environmental challenge. The high water content of potato pulp, typically exceeding 80%, limits its use in animal feed owing to its low nutritional value. Liu, B.N. et al. investigated a novel approach involving the mixed fermentation of potato residue and wastewater, devoid of additional nitrogen sources, to address solid and liquid pollution in the starch industry (Liu et al. 2013). The researchers identified four key microorganisms involved in this process: *Curacaobacter*, *Pseudoalteromonas*, *Paenibacillus*, and *Bacillus*. The resulting SCP products contained 46.09% protein. The potential for industrial applications was demonstrated by scaling up to a 150 m^3^ fermentation system. Moreover, the utilization of waste materials derived from date syrup and cheese byproducts as substrates has demonstrated considerable potential for the generation of high-quality SCP (Yadav et al. 2014; Al-Farsi et al. 2019).

Lignocellulosic biomass, derived from plants through photosynthesis, primarily comprises cellulose, hemicellulose, and lignin. Although it is employed in several industries, including papermaking, construction, textiles, and wood processing, its full potential remains largely untapped (Liu 2010). The bioconversion of lignocellulosic biomass is of paramount importance for the two principal strategies for SCP production: enzymatic hydrolysis followed by yeast fermentation or simultaneous saccharification and fermentation using cellulose and hemicellulose-degrading bacteria and yeast (Zhang et al. 2006). Yeast species have been demonstrated to be effective in utilizing lignocellulosic hydrolysates, which represent the most abundant bioresources for bioproducts. Wu et al. used lignocellulosic hydrolysates and xylose to generate SCP with *Candida intermedia* FL023, using peptone and yeast extract as nitrogen sources, and attained a crude protein content of 484.2 g/kg DCW (Wu et al. 2018). Silva et al. (2003) optimized the nutritional conditions for culturing *Paecilomyces variotii* in eucalyptus hemicellulose hydrolysate by adding ammonium sulfate and sodium phosphate, resulting in a cell concentration of 12.06 g/L (Silva et al., 2003).

These studies underscore the potential of a range of agro-processing byproducts and lignocellulosic biomass as sustainable substrates for SCP production, advancing both environmental sustainability and economic viability across diverse industrial sectors.

### Byproducts of livestock and fisheries

The byproduct of fishmeal processing—water—serves as a substrate for SCP production. In a separate study, Kam et al. employed a combination of *Lactobacillus acidophilus* and *Aspergillus niger* to transform stick water into SCP. Both microorganisms demonstrated effective utilization, with *L. acidophilus* reaching a biomass of 7.29 g/L and *A. niger* reaching 5.20 g/L (Kam et al. 2012). Undigested poultry litter (UPL) and shrimp shell waste have been investigated as potential alternative raw materials for SCP production (Jalasutram et al. 2012; Wu et al. 2018). The resulting SCP products can be used as supplements in animal feed, offering advantages, such as cost reduction and mitigation of waste-related environmental issues.

### One-carbon compounds

One-carbon (C_1_) compounds, including methanol, methane, and carbon dioxide, are ubiquitous in both natural and industrial processes; however, they are frequently under-used. The conversion of these C_1_ compounds into high-value SCPs not only reduces carbon emissions and mitigates the greenhouse effect but also promotes sustainable development (Ritala et al., 2017; FU et al. 2023).

Methanol, a low-cost and abundant raw material, is widely used as a starting material for SCP production. Analysis of the cell composition of *Methylobacterium organophilum* revealed a high crude protein content and the presence of essential amino acids, indicating its potential for use in SCP biomass. Ana et al. cultivated *M. organophilum* using methanol as a carbon source, attaining a maximum DCW of approximately 5 g/L with an initial methanol concentration of 12 g/L and a methanol conversion rate of 0.42 g DCW/g (Simões et al. 2022). *P. pastoris*, which is renowned for its natural methanol assimilation capability, was optimized for methanol-based SCP synthesis via adaptive laboratory evolution (ALE). Meng et al. successfully enhanced methanol utilization efficiency and temperature tolerance in *P. pastoris*, achieving high biomass production (63.37 g DCW/L), a methanol conversion rate of 0.43 g DCW/g, and a protein content of 0.506 g/g DCW in pilot-scale fed-batch cultivation at 33 °C (Meng et al. 2023a).

Methane (CH_4_), a potent greenhouse gas emitted from natural and anthropogenic sources (Jain, 2019), has been harnessed for SCP production using *M. capsulatus* in large-scale fermentation processes by companies such as UniBio and Calysta (Øverland et al. 2010; Zhang et al. 2022). Advances in urban sewage and garbage treatment technologies have enabled the use of methane-oxidizing bacteria to convert methane into SCP. Zha et al. demonstrated the successful growth of a mixed strain comprising 56.26% *Methylomonas* and 24.60% *Methylobacter* on pasteurized anaerobic digestion (AD) supernatant and biogas from sewage sludge, achieving promising dry cell weight yields (0.66 gDCW/gCH_4_ and 11.54 gDCW/gNH^4+^) with a protein content exceeding 41% in the DCW (Zha et al. 2021). Benyamin et al. employed biogas as a substrate for methane-oxidizing bacteria, resulting in high biomass yields (0.87 gDCW/gCH_4_) through the addition of nitrogen from pasteurized centrifuged digestate or electrochemically extracted ammonium from digestate (Khoshnevisan et al., 2019).

Carbon dioxide (CO₂) is a primary greenhouse gas and a key target for energy conservation and emission reduction. Autotrophic bacteria capable of CO₂ fixation are particularly well-suited for the sustainable production of SCP. Photosynthetic cyanobacteria, which use light as an energy source, demonstrate considerable potential for carbon dioxide fixation in SCP (Hülsen et al. 2022). They offer notable advantages over plant-based approaches, including rapid growth and versatile application potentials.

## Engineering strategies for enhancing SCP production

Microbial biomass yield and protein content are of paramount importance for influencing SCP production. An increase in microbial biomass yield has been demonstrated to enhance SCP productivity, whereas an improvement in protein content has been shown to elevate the nutritional quality of SCP. Consequently, considerable effort has been invested in the pursuit of increasing microbial biomass and protein content to enhance SCP production.

### Strategies to enhance microbial biomass yield

**Fig. 2:**
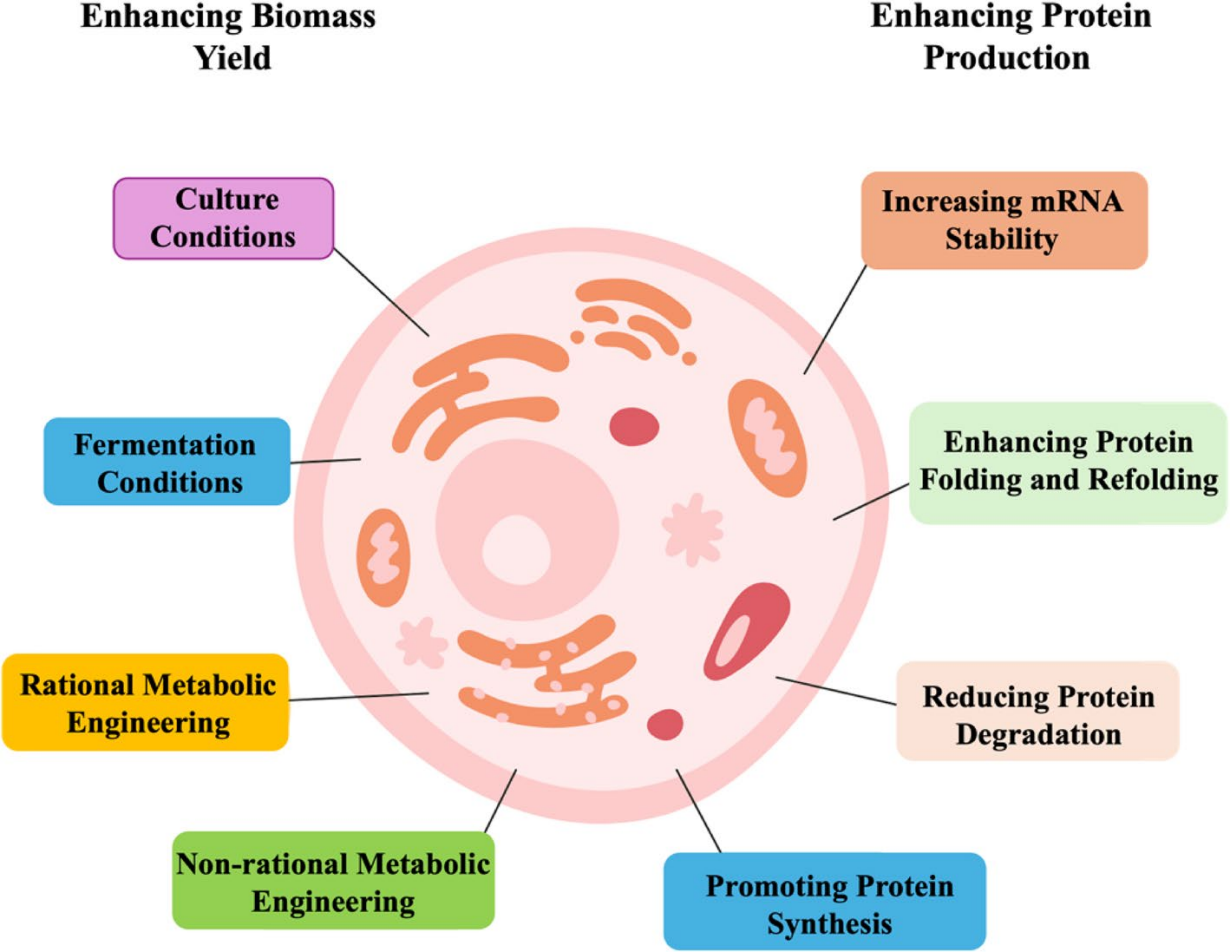
Metabolic engineering for enhancing biomass and protein yield in SCP production

Microbial growth is a critical factor influencing the production of SCP, as it directly affects the biomass yield and protein content. Efficient microbial growth is a prerequisite for a high biomass output, which is a necessary condition for cost-effective SCP production. Several factors influence microbial growth, including nutrient availability, presence of toxic compounds, substrate tolerance, cultivation conditions, and metabolic constraints (Reihani and Khosravi-Darani, 2019). Various strategies are being used to address these challenges and enhance microbial growth to improve the protein content in SCP production (Balagurunathan et al., 2022) (Fig. 2).

#### Optimization of culture conditions and fermentation processes

The production of microbial biomass has been enhanced by optimizing culture conditions (Tesfaw and Assefa, 2014; Hezarjaribi et al., 2016; Kerckhof et al. 2021; Vethathirri et al. 2021; Hu et al. 2022) and improving fermentation processes to achieve high-protein yields (Reihani and Khosravi-Darani, 2019). For example, optimization of the culture medium for *S. cerevisiae* using the *Taguchi method*, which adjusts the concentrations of ammonium sulfate, iron sulfate, glycine, and glucose based on signal-to-noise ratio analysis, has been demonstrated to significantly improve SCP production. Before optimization, the yield was 7.69–7.91 log colony-forming unit (CFU) mL^−1^ with a protein content of 34% DCW. Following optimization, the yield increased to 8.84 log and the protein content reached 44.6% (Hezarjaribi et al. 2016). Research on the optimization of microbial biomass production through co-culture methods has demonstrated notable improvements in both the biomass yield and protein content of SCP. For instance, the co-culturing of white-rot fungi (*Ganoderma lucidum*) with yeast (*Candida utilis*) resulted in a notable increase in protein content from 8.75% to 16.23%. Similarly, the combination of *Trichoderma reesei* and *A. niger* resulted in a 91% increase in biomass yield, from 11.2 g/L to 21.4 g/L (Tesfaw and Assefa, 2014). Subsequent research demonstrated that the co-cultivation of *Xanthobacter variabilis* and *Shinella* sp. NM-101 resulted in an 11.4-fold increase in bacterial growth, along with a 24% increase in protein content, 28% increase in essential amino acids, and 26% increase in overall biomass yield compared to monocultures (Hu et al. 2022). The co-cultivation of distinct microorganisms facilitates the collaborative utilization of carbon sources, breakdown of complex substrates, and removal of deleterious metabolic byproducts.

The yield and quality of SCP production are significantly influenced by fermentation parameters, including carbon and nitrogen sources, inoculum size, age, aeration, temperature, and pH. These parameters play a crucial role in optimizing microbial growth and metabolic activity, which affects the yield and quality of SCP production. It has been demonstrated that adjusting these parameters is crucial for maximizing biomass yield, enhancing productivity, reducing production costs, and improving SCP quality (Reihani and Khosravi-Darani, 2019).

#### Rational metabolic engineering approaches

Metabolic engineering, encompassing both rational and non-rational engineering methodologies, is of paramount importance for enhancing SCP production. Modifying microbial metabolic pathways can enhance substrate utilization efficiency and increase nutrient conversion efficiency, improving overall biomass and protein yield (Jang et al., 2012).

Rational engineering approaches involve modification of transcription factors, transporters, and metabolic pathways to achieve the desired phenotype (Hara et al. 2017; Gupta et al. 2020). For example, optimization of transporter proteins in yeast, such as glucose transporters (Hxt1p and Hxt7p), sucrose transporters (Agt1p), cellodextrin transporters (CDT-1), xylose transporters (Hxt7p), and galactose transporters (Gal2p), has been demonstrated to significantly enhance substrate utilization efficiency (Hara et al., 2017). The overexpression of Hxt7p resulted in enhanced glucose consumption and lactate production, whereas the introduction of CDT-1 from *Neurospora crassa* led to a 30%–50% increase in sugar utilization rates and a 20%-40% increase in ethanol yield (Hara et al., 2017). These transporter engineering strategies optimize the bioproduction processes in yeast, demonstrating their potential for industrial applications in SCP production. Furthermore, global transcription machinery engineering (gTME) has yielded notable advancements in microbial biomass and product yields (Tan et al. 2016; El-Rotail et al., 2017). For instance, the implementation of gTME has demonstrated a remarkable enhancement in the biomass and ethanol production capacity of *S*. *cerevisiae*. The mutants exhibited a notable enhancement in DCW, reaching 2.01 g/L in 3% ethanol, compared to the control strain, which demonstrated a DCW of 1.39 g/L. Moreover, the most efficient mutant exhibited an ethanol yield of 15.72 g/L, representing a 60.3% increase over that of the control strain (9.814 g/L) (El-Rotail et al., 2017). Similarly, the *Zymomonas mobilis* mutant strain ZM4-mrpoD4 exhibited enhanced growth with a maximum cell density (OD_600_) of approximately 1.8 compared to the control's 1.2 under 9% ethanol stress, and increased ethanol production from 6.6–7.7 g/L to 13.0–14.1 g/L (Tan et al. 2016). Metabolically engineered *S. cerevisiae* strains were cultivated in ethylene glycol, isopropanol, and propionic acid to produce glucose. The protein content of these genetically modified strains reached approximately 50% of their dry cell weight (Tang et al. 2023).

The optimization of carbon fixation and nitrogen assimilation pathways is a critical strategy for enhancing microbial biomass. For example, in *Methylophilus* methylotrophus, overexpression of genes involved in carbon fixation, such as *rbcL* (encoding the RuBisCO large subunit) and *prkA* (encoding phosphoribulokinase), can enhance the flow of carbon into biomass production. Concurrently, overexpression of genes involved in nitrogen assimilation, such as *gdhA* (encoding glutamate dehydrogenase) and *glnA* (encoding glutamine synthetase), has been shown to enhance the availability of amino acids essential for protein synthesis. These modifications have increased protein content by up to 25% and biomass yield by approximately 20%, contributing to a higher SCP output (Windass et al., 1980).

Another crucial strategy is to minimize byproduct formation and carbon loss. In *F. venenatum*, research has concentrated on the inhibition of the ethanol synthesis and gluconeogenesis pathways, which are the primary routes for byproduct formation. Deletion of the genes *FvPDC6* (pyruvate decarboxylase), which is involved in ethanol production, and *FvPCK* (phosphoenolpyruvate carboxykinase), which is involved in gluconeogenesis, whereas the overexpression of *FvPYC* (pyruvate carboxylase) to enhance carbon fixation and optimize the fermentation medium with the addition of ZnSO, resulted in an improvement in the carbon conversion ratio by up to 73% and an increase in protein content by 25%, reaching a protein concentration of 61. This equates to 9% DCW. Furthermore, the synthesis rate increased by 57% and CO₂ emissions were reduced by 39%, thus rendering the process both efficient and environmentally friendly (Tong et al., 2024).

#### Non-rational metabolic engineering approaches

Besides these rational engineering techniques, non-rational engineering approaches, including ALE, chemical mutagenesis, and genome engineering, have been employed for biomass accumulation by improving cell growth and substrate tolerance (Pham et al., 2017; Bennett et al. 2021; Meng et al. 2023). For example, a pH-sensing riboswitch system with a self-regulating genetic program enables bacteria to evolve autonomously, resulting in strains with enhanced survival rates ranging from 20 to 40% in the presence of various organic acids (fumaric acid, citric acid, L-malic acid, itaconic acid, lactic acid, and succinic acid) compared to the control strain T10 (Pham et al. 2017). The combination of ALE with chemical mutagenesis using N-methyl-N'-nitro-N-nitrosoguanidine (NTG) has resulted in notable improvements in the tolerance of mutant strains to methanol. The resulting strains, which exhibited mutations in the 30S ribosomal subunit proteins, particularly in *rpsQ* and *rpsL*, demonstrated growth rate improvements of 38% with 2 M methanol, 57% with 3 M methanol, and 10% with 1 M methanol. In addition, they exhibited a two- to three-fold improvement in methanol utilization at 60 mM (Bennett et al. 2021). Moreover, recent developments in methanol-used yeast *P.* pastoris have demonstrated considerable potential for sustainable SCP production. By employing the ALE approach and implementing genetic modifications, the engineered strain HTX-33 demonstrated a biomass yield of 63.37 g DCW/L, a methanol conversion rate of 0.43 g DCW/g, and a protein content of 0.506 g/g DCW at 33 °C. Compared with the parent strain X-33, which exhibited a protein content of 0.364 g/g DCW and a total nitrogen content of 0.058 g/g DCW at 30 °C, these findings indicate a 39% increase in protein content and a 25.86% increase in total nitrogen (Gao et al., 2023; Meng et al. 2023). These improvements highlight the potential of genetically modified *P. pastoris* strains for efficient SCP production.

### Strategies to enhance protein content of microbial biomass

Besides increasing microbial biomass to enhance protein yield, current metabolic engineering strategies focus on directly boosting protein production. These strategies encompass an array of approaches, including enhancement of mRNA stability, reduction of protein degradation, promotion of protein synthesis, and facilitation of protein folding and refolding (Balagurunathan et al., 2022; see Fig. 2).

#### Increasing mRNA stability

The reduction of mRNA degradation can be achieved through genetic engineering, which allows for the development of RNase-deficient microbes, optimization of mRNA sequence elements, and regulation of RNA-binding proteins. These strategies collectively enhance mRNA stability and protein synthesis, increasing the SCP production in microbial systems. The degradation of mRNA is influenced by several factors, including the sequence of mRNA, activity of RNA-binding proteins, and presence of ribonucleases (Hui et al. 2014; Roux et al., 2022). To reduce total mRNA degradation, a direct method involves the creation of ribonuclease (RNase)-deficient microbes through genetic engineering techniques, such as CRISPR-Cas9-mediated knockout or inhibition of key RNase gene expression, such as the *RNase E* gene. These modifications have been demonstrated to significantly extend mRNA half-life by decreasing mRNA decay rates, improving mRNA stability, and increasing overall mRNA availability for translation, consequently enhancing protein synthesis (Roux et al., 2022). For example, *E. coli* strains, such as BL21 Star™(DE3), with the rne-131 allele for truncated *RNase E* have been demonstrated to be effective in stabilizing mRNA and enhancing heterologous protein synthesis. The use of RNase-deficient strains has been shown to increase protein yields. This is evidenced by strategies such as MazF induction, which has been shown to boost protein levels by a factor of 3–11 (Venturelli et al. 2017; Wu et al. 2020).

It is crucial to optimize the sequence elements of mRNA to ensure stability. Modifications to the 5' untranslated region (UTR) through the removal of destabilizing sequences and addition of stabilizing elements protect mRNA from degradation, enhance ribosome binding, and improve heterologous protein expression. For example, incorporating hairpin structures derived from *ompA* resulted in an increase in the mRNA half-life from three minutes to 5–6 min. Further addition of an AU-rich element extends this to 12.9 min (Viegas et al. 2018). The introduction of synthetic REP sequences at the 3′-terminus of prokaryotic cells prevents 3′-5′ exonuclease degradation, significantly improving heterologous protein expression. For example, the activity of cyclodextrin glucosyltransferase (CGTase) increased from 210.6 to 291.5 U/mL, whereas glucosamine-6-phosphate *N*-acetyltransferase 1 (GNA1) activity rose from 524.8 to 890.7 U/mg (Deng et al. 2019). Furthermore, differential mRNA decay within bacterial operons, such as the tatABC operon, highlights the influence of stabilizing RNA elements on protein synthesis. Stabilizing tatA mRNA over tatBC has been demonstrated to results in 25-fold higher TatA protein expression (Dar and Sorek, 2018). Another effective strategy involves the regulation of RNA-binding proteins. Proteins that stabilize mRNA, such as HuR, bind to mRNA and protect it from degradation. Conversely, destabilizing proteins, such as tristetraprolin, reduce mRNA stability by increasing mRNA decay rates. Therefore, mRNA stability can be enhanced by reducing the expression or activity of destabilizing proteins (Mukherjee et al., 2011).

#### Promoting protein synthesis

The construction of an efficient, high-expression system through codon optimization, increase in gene copy number, and transcriptional regulation is of paramount importance for achieving high-protein yields. Codon optimization enhances translation efficiency by adjusting codon usage frequencies, whereas increasing the gene copy number involves the integration of multiple copies of the target gene into plasmids or the genome. For example, codon optimization has been employed to replace rare codons in the native *T. emersonii* α-amylase gene with codons preferred by *S. cerevisiae*, resulting in a 1.6-fold increase in protein yield. Similarly, codon optimization of the *T. emersonii* glucoamylase gene resulted in a 3.3-fold increase in the protein yield (Cripwell et al., 2019). The use of high-copy number 2μ-based YEp plasmids, which can maintain 10–40 copies, has demonstrated a significant enhancement in protein expression, with yields of up to 5 g/L for human albumin and albumin fusion proteins (Chen et al.). 2012; Da Silva and Srikrishnan 2012). Transcriptional regulation uses strong promoters, inducible promoters, synthetic promoters, and optimized terminator sequences to enhance transcriptional and translational efficiency. Strong constitutive promoters in *S. cerevisiae* include *pTDH3*, *pPGK1*, *pADH1*, and *pTPI1* as well as inducible promoters such as *pGAL1*, *pGAL7*, *pGAL10*, *pPHO5*, and *pMET25* (Zhao et al., 2024). The *GAP1* promoter system, with a medium-switching strategy, yielded approximately 40 g/L DCW and 1 mg/L Gap1 protein, increasing target protein production by approximately five-fold compared with the PMA1 promoter system (Debailleul et al., 2013). With the advent of synthetic biology, high-activity synthetic promoters with broad activity ranges have been developed (LaFleur et al., 2022). Synthetic promoters have been used to enhance the production of green fluorescent proteins and β-glucosidase in *S. cerevisiae* (Deng et al., 2021; Wu et al. 2023). The use of the inducible MET25 promoter in methionine-free media resulted in a significant enhancement in the expression of target proteins, with an 18- to 70-fold increase observed during the 16 to 24-h period compared to conditions with methionine (Solow et al., 2005). Moreover, the implementation of these strategies integratedly has been demonstrated to significantly enhance heterologous protein production in *S*. *cerevisiae*, providing effective solutions for industrial-scale applications (Zhao et al., 2024).

#### Enhancing protein folding and refolding

Enhancement of protein folding and refolding represents an effective strategy for increasing protein production, whereby reduction of protein degradation due to misfolding is achieved. Proteasomes or autophagy pathways are responsible for the degradation of properly folded proteins, increasing the net protein yield (Díaz-Villanueva et al., 2015). Molecular chaperones such as Hsp70 and Hsp90 facilitate the correct folding of proteins by binding to unfolded proteins and preventing their aggregation (Taldone et al. 2014). Overexpression of the chaperone protein BiP or disulfide isomerase Pdi1p has been demonstrated to enhance the production of β-glucosidase, endoglucanase, and α-amylase (Tang et al., 2015). Significant improvements in protein production efficiency have also been reported (Kim et al. 2020). The co-expression of chaperones and foldases has demonstrated a significant enhancement in protein yields through the optimization of refolding conditions. For example, the helper plasmid *pTUM4*, which overexpresses four periplasmic folding catalysts (DsbA, DsbC, FkpA, and SurA), has been demonstrated to significantly enhance the folding efficiency of recombinant proteins, increasing the yield of soluble proteins. Specifically, the use of *pTUM4* in the production of human plasma retinol-binding protein (RBP) resulted in a four-fold increase in the yield of correctly folded RBP, reaching 0.6 mg/L/OD (Schlapschy et al. 2006). Furthermore, the expression of genes involved in the unfolded protein response (UPR) pathways, including *HA*C_1_, *ATF6*, *PERK*, and *IRE1*, has been shown to enhance folding capacity and mitigate folding stress induced by misfolded proteins (Smith et al. 2011; Chambers & Marciniak, 2014). Overexpression of *HA*C1 enhances the production of a range of recombinant proteins, including xylanase and α-amylase (Valkonen et al., 2003; Bao et al., 2020). Moreover, chemical chaperones such as 4-phenylbutyrate (4-PBA) and tauroursodeoxycholic acid have been shown to stabilize protein folding and reduce aggregation, representing a potential strategy for improving protein content (Lindquist and Kelly, 2011).

#### Reducing protein degradation

The most direct method for enhancing the protein yield is to inhibit protein degradation via protease knockout. The study deleted eight protease genes (*nprE*, *aprE*, *epr*, *bpr*, *mpr*, *nprB*, *vpr*, and *wprA*) were identified in *Bacillus subtilis*, resulting in second-generation protease-deficient strains. The engineered strain PD8, which was created by eliminating eight detrimental proteases, demonstrated notable improvements in methyl parathion hydrolase (MPH) production (79.9 U/mL in PD8 vs. 32.5 U/mL in the WB800-control strain) and chlorothalonil hydrolytic dehalogenase (Chd) production (13.4 U/L in PD8 vs. 7.4 U/L in WB800) (Zhao et al., 2019). Another study investigated the potential of protease-deficient *S. cerevisiae* strains to produce human-compatible glycoproteins. Disruption of the *PEP4* and *PRB1* genes in the resulting protease-deficient strain YAB101-4 resulted in a tenfold increase in human interferon-β (hIFN-β) secretion compared to the unmodified strains (Tomimoto et al., 2013). These studies demonstrate considerable potential for improving foreign protein production through the use of protease knockout strategies.

The ubiquitin–proteasome system and autophagy pathways play crucial roles in the degradation of misfolded proteins, maintaining proteostasis (Varshavsky, 2017). Spatial compartmentalization mechanisms such as juxtanuclear quality control (JUNQ) and insoluble protein deposition (IPOD) sequester misfolded proteins, facilitating proper folding or degradation (Kaganovich et al. 2008; Wolff et al. 2014). Despite the limited attention these mechanisms have received in the context of protein production, their optimization has the potential to enhance the protein content in SCP production.

## Industrial application of SCP

The high-protein content and nutritional richness of SCP, coupled with its sustainable production capabilities, make it a suitable candidate for a plethora of industrial applications, including food, animal feed, and feedstock for microbial fermentation (Onyeaka et al., 2022; Fact.MR, 2023).

### Food applications

SCP is a widely used ingredient in the food industry, largely because of its high-protein content and nutritional value. This provides a sustainable alternative to traditional protein sources. One notable application of SCP is the production of high-protein foods and beverages, which are especially advantageous for individuals who require a high-protein diet, such as vegetarians and athletes. SCP has been incorporated into a variety of food products, including protein bars, drinks, and powders, and is a rich source of essential amino acids (Onyeaka et al. 2022; Fact.MR 2023). Solar Foods produce Solein®, a protein-rich product with a content of 65%-70% DCW, derived from *Xanthobacter tagetidis* using electricity and carbon dioxide. Solein is used in a variety of food products, including protein shakes, bread, and pasta (Solar Foods n.d.). Furthermore, SCP serves as a principal component in the production of meat substitutes, imitating the texture and flavor of meat while reducing its environmental impact. Examples of such products include plant-based burgers, vegetarian sausages, and chicken substitutes (Bratosin et al. 2021). Nature's Fynd offers Fy Protein™, a high-protein ingredient (> 50%) derived from fungal microbes, which is used in the production of products such as meatless breakfast patties and dairy-free cream cheese (Nature's Fynd n.d.). Similarly, Quorn employs *F. venenatum* fermentation technology to produce mycoproteins, which are incorporated into a diverse array of vegetarian and plant-based foods, including Quorn Meatless Grounds and Quorn Meatless Nuggets (Quorn n.d.). Approximately 50% of DCW comprises mycoproteins. Furthermore, SCP is employed in the production of fermented foods, such as yogurt and tofu, to enhance their nutritional value and flavor while extending their shelf life. For example, Perfect Day employs microbial fermentation technology to create Perfect Day Dairy Protein™, a protein substitute incorporated into products such as Brave Robot ice cream and Modern Kitchen cream cheese. This enhances the nutritional value and flavor of these products (Perfect Day n.d.).

SCP is a valuable source of essential amino acids, vitamins, and other nutrients, making it an optimal ingredient for the production of dietary supplements, functional foods, and bioactive compounds for therapeutic treatments. These products benefit from the rich protein content and essential nutrients of SCP, which are crucial for maintaining a balanced diet and preventing nutrient deficiencies (Onyeaka et al., 2022; Fact.MR, 2023). The fermentation process yielded Blue Origins®, an SCP product derived from microalgae. This product is rich in omega-3 fatty acids and is used in dietary supplements and functional foods to promote cardiovascular health (Fermentalg n.d.). Solazyme developed AlgaVia®, an SCP that is rich in protein and essential nutrients. It is used in a range of nutraceutical products to enhance their health benefits (TerraVia n.d.). Therefore, SCP is a promising alternative protein source for the food industry.

### Feed applications

SCP has been extensively used as a valuable alternative to traditional protein sources such as soybean meal and fishmeal in animal feed applications, including poultry, aquaculture, and livestock feed. This is because of its high-protein content, balanced amino acid profile, and sustainable production methods. In poultry feed, incorporation of SCP has been demonstrated to enhance growth rates and improve overall health. This is attributed to its contribution to improved feed efficiency and the production of higher-quality meat (Onyeaka et al., 2022; Fact.MR, 2023). For example, Unibio employs *M. capsulatus* to manufacture UniProtein®, an SCP with a protein content of 70% of the DCW. This product is derived from methane and is used in poultry feed, offering a sustainable and high-quality protein alternative (Unibio n.d.). SCP provides the essential nutrients required to support the growth and health of fish and shrimp, conferring significant benefits to aquaculture through the use of SCP-based aquaculture feed. FeedKind® by Calysta is produced through the fermentation of *M. capsulatus* using natural gas, resulting in an SCP with a protein content of 80% of the DCW. It is frequently used as an aquaculture feedstuff and offers notable sustainability benefits (Calysta n.d.). Furthermore, SCP is used in livestock feed to enhance protein intake and facilitate the growth of cattle, swine, and other farm animals. The utilization of SCP in livestock feed mitigates the environmental impact of animal husbandry, while ensuring a consistent and reliable supply of high-quality protein. KnipBio produces KnipBio Meal (KBM), an SCP derived from *M. extorquens*, which is used in livestock feed to enhance feed conversion rates and promote healthy animal growth (KnipBio n.d.). These applications in animal feed illustrate the potential for SCP to provide environmental and economic benefits to the livestock, poultry, and aquaculture industries.

### Feedstock applications

SCP is a rich source of protein, making it a frequently used feedstock for microbial fermentation for the production of a range of valuable products, including bioplastics and biofuels, which have high protein and carbohydrate contents. For example, NovoNutrients produce NovoMeal, an SCP derived from industrial carbon dioxide emissions. NovoMeal™ is an exemplary fermentation feedstock for the production of sustainable protein ingredients that are highly digestible and exhibit superior amino acid profiles compared with numerous conventional protein sources. These proteins are employed in animal feed, plant-based foods, and aquaculture feed, underscoring the versatility and environmental benefits of utilizing SCP in fermentation processes (NovoNutrients n.d.). Mango Materials is another company that employs methane-derived SCP to manufacture polyhydroxyalkanoates (PHA), a biopolymer used in the production of biodegradable plastic products, including packaging, agricultural films, and consumer goods (Mango Materials n.d.). Furthermore, SCP has been successfully employed for biofuel production. For example, LanzaTech employs carbon-rich industrial emissions to cultivate *C. autoethanogenum*, yielding SCP with 80% DCW protein content. Subsequently, SCP is converted into bioethanol and other valuable products via fermentation (Ma et al., 2022). This process not only results in a reduction in greenhouse gas emissions but also produces a renewable biofuel that can be used as an alternative to traditional gasoline. Algenol produces biodiesel from SCP derived from the microalga *Euglena gracilis*, which has a protein content exceeding 70% DCW. The process exploits the high lipid content of microalgae to create a sustainable alternative fuel source while simultaneously producing a nutrient-rich protein byproduct (Algenol n.d.). SCP proves to be the ideal feedstock for bioplastics, biofuels, and efficient production of valuable chemicals.

## Shortages and safety concerns of SCP production

SCP production offers substantial environmental and nutritional advantages by transforming agricultural and industrial waste into high-protein biomass, thus advancing sustainability and mitigating the environmental consequences associated with conventional protein sources (Ritala et al., 2017). Microorganisms, including algae, fungi, bacteria, and yeast, facilitate the efficient production of SCP with rapid growth rates and high yields of proteins enriched with essential amino acids, vitamins, and minerals (Aidoo et al. 2023). Nevertheless, the production and consumption of SCP have certain shortcomings and safety concerns (Flight et al. 2024).

A noteworthy concern is the elevated nucleic acid content of SCP, particularly in bacteria and yeasts. This can cause uric acid accumulation in humans, which may lead to adverse health outcomes such as gout and kidney stones (Yang et al. 1979; Ravindra, 2000). To mitigate these risks, processes such as thermal treatment, chemical extraction, and enzymatic degradation can be used to reduce nucleic acid levels. Heat treatment can denature nucleic acids, rendering them less bioavailable, thus reducing the risk of uric acid accumulation (Onyeaka et al., 2022).

Certain microorganisms involved in SCP production can produce harmful endotoxins and mycotoxins, posing a toxic risk. To address this issue, the selection of non-toxigenic strains and the application of genetic modifications can help to minimize the presence of these toxins (Ravindra, 2000; Hadi and Brightwell 2021). It is of paramount importance to implement robust regulatory frameworks and quality control measures to guarantee that SCP products comply with requisite safety standards. For example, the European Union regulations on novel foods and the Food and Drug Administration (FDA) guidelines for food safety set rigorous standards for the production and quality control of SCP products (Council, 2015).

The digestibility and bioavailability of SCPs are of great consequence to human health, necessitating a comprehensive evaluation through clinical studies to ensure their suitability for consumption. For example, research on the digestibility of algal proteins has demonstrated that processing techniques can enhance bioavailability and nutritional quality (Junaid et al. 2020; Bratosin et al. 2021).

It is imperative to address consumer neophobia and potential allergic reactions to foster the acceptance of SCP as an alternative protein source. Consumers’ acceptance of SCP products is a crucial factor in determining their success (Hung et al. 2023). The public's perception of SCPs can be shaped by several factors, including awareness of their environmental benefits, trust in their safety and nutritional value, and cultural attitudes toward novel food sources (Siddiqui et al. 2022). Effective communication strategies, transparency in production processes, and educational campaigns are essential to foster consumer trust and acceptance. Implementing public education campaigns and transparent communication on the safety and benefits of SCP can facilitate the establishment of consumer trust. Moreover, allergenicity testing is crucial for identifying and mitigating potential allergic reactions (Siddiqui et al. 2022; Hung et al. 2023).

In conclusion, whereas SCP production offers a sustainable solution with substantial benefits, it is imperative to address safety and risk factors through optimized processes and rigorous regulatory oversight. This information is crucial for establishing SCP as a safe and viable alternative protein source for the global food system. By employing advanced biotechnological methods, adhering to strict regulatory standards, and engaging in comprehensive safety evaluations, SCP can contribute to sustainable protein production and food security.

## Data Availability

No datasets were generated or analysed during the current study.
